# High-altitude environments elevate central and peripheral α-synuclein levels and accelerate PD-related pathology

**DOI:** 10.1186/s12967-026-09103-z

**Published:** 2026-10-21

**Authors:** Hao-Lun Sun, Dong-Yu Fan, Jun-Hao Xie, Qing Du, Rui-Jie Zeng, Jian-Ni Hu, Jun Liang, Li Zhu, Xu Wang, Hao-Xiang Wang, Qing Zhao, Rong Zhang, Yan-Jiang Wang, Hong-Da Zhao, Wang-Sheng Jin

**Affiliations:** 1 Department of Neurology and Centre for Clinical Neuroscience, Daping Hospital, Army Medical University, Chongqing, China; 2 Chongqing Key Laboratory of Ageing and Brain Diseases, Chongqing, China; 3 Institute of Brain and Intelligence, Army Medical University, Chongqing, China; 4 Department of Special Medicine, Daping Hospital, Army Medical University, Chongqing, China; 5 Shigatse Branch, Xinqiao Hospital, Army Medical University, Shigatse, China; 6 Department of Neurology, The Second Affiliated Hospital, Xinqiao Hospital, Army Medical University, Chongqing, China; 7 Department of Neurology, The 910th Hospital of the Joint Logistics Support Force of the Chinese PLA, Quanzhou, Fujian Province China; 8 Department of Neurology, Army 73rd Group Military Hospital of the Chinese PLA, Xiamen, Fujian Province China; 9 No. 10 Changjiang Branch Road, Yuzhong District, Chongqing, China; 10 Tibet Autonomous Region, No. 5 Zhufeng West Road, Shigatse, China

**Keywords:** High altitude, Parkinson’s disease, Alpha-synuclein, Hypoxia, Erythrocyte

## Abstract

**Background:**

High-altitude exposure has been linked to an increased risk of Parkinson’s disease (PD), potentially through hypoxic neuronal damage, yet the involvement of α-synuclein (α-syn) remains poorly understood. This study integrates population-based surveys, animal experiments, and clinical cross-sectional data to elucidate the ro*le* of high-altitude environments in PD pathogenesis.

**Methods:**

A cross-sectional study was conducted among middle-aged and elderly high-altitude residents. Additional cohorts (low-altitude PD patients, low-altitude controls, and high-altitude controls) were enrolled for plasma and erythrocytic α-syn measurement, and a subset of PD patients underwent transcranial sonography to assess correlations between erythrocyte indices and substantia nigra echogenicity. A53T transgenic mice were exposed to high altitude for six months and evaluated by behavioral tests, omics sequencing, α-syn assays, and immunohistochemistry. Erythrocyte-derived extracellular vesicles from PD patients were intravenously injected into wild-type mice to assess brain entry.

**Results:**

The crude prevalence of PD tended to be higher in high-altitude populations than the national average. A53T mice exposed to high altitude displayed more severe motor deficits, elevated brain α-syn levels, and pronounced dopaminergic neuron loss. Omics analyses revealed enrichment of pathways related to IL-17, sphingosine-1-phosphate signaling, and oxidative stress in brain tissue from high-altitude versus low-altitude A53T mice, suggesting blood-brain barrier disruption and impaired α-syn clearance. In peripheral blood, both high-altitude mice and humans exhibited elevated α-syn levels compared with their low-altitude counterparts, and erythrocyte indices correlated positively with α-syn levels. After normalization to erythrocyte count and hemoglobin, however, erythrocytic α-syn did not differ between high-altitude and low-altitude controls. In mice, intravenously injected human-derived erythrocytic vesicles entered the brain.

**Conclusions:**

Chronic high‑altitude exposure is associated with peripheral and central α‑syn accumulation, which may be driven by a hypoxia‑induced erythrocyte pool expansion and increased α-syn burden, potentially aggravating PD‑related neuropathology.

**Supplementary Information:**

The online version contains supplementary material available at https://doi.org/10.1186/s12967-026-09103-z.

## Background

Parkinson’s disease (PD) is a neurodegenerative disorder that predominantly affects elderly people and is characterized by cardinal motor symptoms, including bradykinesia, resting tremor, and postural instability [1]. As the fastest-growing neurological disease globally, the PD projected prevalence is expected to surpass 25 million cases by 2050 [2]. Central to the pathogenesis of PD is the aberrant aggregation of alpha-synuclein (α-syn), whose hyperphosphorylated form leads to the formation of Lewy bodies, driving dopaminergic neurodegeneration [3]. Recent evidence suggests that peripheral α-syn may infiltrate the central nervous system, potentially initiating neuropathological cascades [4, 5].

The pathological mechanisms described above were identified under conventional conditions, and the specific mechanisms of PD in high-altitude environments remain unexplored. Notably, approximately 140 million people worldwide reside above 2,500 m [6]. Owing to limited healthcare resources and diagnostic challenges, current epidemiological data likely underestimate the disease burden of PD in high-altitude regions [7]. High-altitude environments, characterized by hypobaric hypoxia, represent a well-established model of chronic hypoxia [8]. Likewise, obstructive sleep apnea syndrome (OSAS) and chronic obstructive pulmonary disease (COPD), two prevalent disorders causing systemic hypoxia, are both associated with increased PD risk, implicating chronic hypoxia in neurodegeneration [9, 10]. Hypoxia-induced oxidative stress and mitochondrial dysfunction may contribute, although this requires further study [11]. The present study aims to combine population-based investigations, animal models, and cross-sectional clinical research to clarify the influence of high-altitude environments on PD pathogenesis.

## Methods

### Epidemiological investigation and PD diagnosis

From January to June 2022, we administered a cross-sectional survey targeting middle-aged and elderly populations (≥ 45 years) in rural communities of Shigatse City, Tibet Autonomous Region (altitude: 3,800 m). A total of 665 eligible participants were enrolled. Standardized questionnaires captured demographic characteristics and medical history.

The clinical diagnosis of PD was based on the 2015 Movement Disorder Society Clinical Diagnostic Criteria [12]. All diagnostic assessments were performed by two neurologists to ensure diagnostic reliability.

### Study subjects

One hundred seventeen patients diagnosed with PD were recruited at Daping Hospital, Chongqing, between January 2021 and December 2023. During this period, we concurrently recruited 115 age- and sex-matched low-altitude controls from the health examination center of the same hospital, along with 106 high-altitude subjects from the Shigatse Branch of Xinqiao Hospital (altitude: 3,800 m). All high-altitude participants had resided at an altitude ≥ 2,500 m for at least three years. The low‑altitude controls resided below 800 m with no chronic hypoxic exposure. Previous studies have demonstrated that ultrasound hyperechogenicity of the substantia nigra (SN) might reflect the severity of PD [13]. Thus, between January 2023 and December 2025, an additional group of 181 PD patients exhibiting SN hyperechogenicity on transcranial sonography was systematically recruited for further analysis. Finally, we recruited 12 high-altitude participants from Shigatse and 11 low-altitude controls from Chongqing to collect erythrocytes in February 2025.

Subjects were excluded for the following reasons: (1) a family history of PD; (2) other concomitant neurological disorders, severe cerebrovascular disease, or Parkinsonism; (3) severe cardiac, pulmonary, hepatic, renal diseases or any kind of tumor; (4) hematologic diseases or other diseases that could affect erythrocytes; (5) recent transfusion history; or (6) any acute illnesses (e.g., infections, diarrhea). Our study was approved by the Institutional Review Board of Daping Hospital (2024 − 394) and Shigatse Branch, Xinqiao Hospital (2023-004) without any reservations. This study was also conducted in accordance with the principles of the Declaration of Helsinki.

### Blood sampling and measurement

Fasting blood was collected between 06:00 am and 07:00 am to avoid potential circadian rhythm effects. The blood samples were centrifuged within one hour after collection and then stored at − 80 °C until use. We obtained informed consent from each participant before blood sample collection. Sample collection and processing followed our group’s previously published standardized protocol described in the Chinese guideline for clinical application of fluid biomarkers for Alzheimer’s disease (2024 edition) [14]. No visible hemolysis was observed during sample processing. Red blood cell count (RBC) and hemoglobin (HGB) were measured with an automatic blood analyzer at the Clinical Laboratory of Daping Hospital. Additionally, peripheral oxygen saturation (SpO₂) was measured using fingertip pulse oximetry (Yuyue YX306, Yuyue Medical Equipment and Supply, China) in high-altitude participants.

To obtain purified erythrocyte extracts, the samples underwent an initial centrifugation at 4 °C (2,000 × g) for 10 min. The upper plasma layer and intermediate buffy coat were meticulously aspirated. The remaining erythrocyte pellet was then resuspended in ice-cold phosphate-buffered saline (PBS). Processed RBC samples were cryopreserved at − 80 °C. The cryopreserved RBC samples underwent two complete freeze‒thaw cycles at room temperature to induce lysis. The lysates were clarified by high-speed centrifugation (20,000 × g, 30 min, 4 °C) to effectively remove cellular debris. Total protein concentrations in the erythrocyte lysates were determined using a BCA protein quantification assay.

Total α‑synuclein levels in plasma and erythrocyte lysates were measured with a human total alpha‑synuclein ELISA kit (BioLegend, 448607). Phosphorylated α‑synuclein (p‑α‑syn) was detected in both sample types using a human phosphorylated alpha‑synuclein ELISA kit (Dakewe, F10455-A). For erythrocytes, final α‑synuclein concentrations were normalized to total lysate protein and expressed as ng/mg. Plasma α‑synuclein levels were expressed as ng/mL. All samples were measured in strict accordance with the manufacturer’s instructions.

### Transcranial sonography

Transcranial sonography (TCS) examinations were performed via a 2 MHz phased-array transducer (Philips Affiniti 70) through bilateral transtemporal bone windows to visualize the midbrain plane. The SN hyperechogenic area was manually delineated, with simultaneous measurement of the midbrain cross-sectional area. The ratio of the SN hyperechogenic area to the midbrain area was calculated. All TCS examinations were performed in the morning during the medication-on state, and patients with severe large-vessel stenosis were excluded. For further analysis, we additionally collected data on disease duration, levodopa-equivalent daily dose (LEDD), and Hoehn & Yahr (H&Y) stage from the patients who underwent TCS. LEDD was calculated according to the updated systematic review [15]. All procedures were conducted by an experienced technician and checked by a senior doctor.

### Animal design and behavioral assays

A53T transgenic mice and Wt mice were obtained from Nanjing MiceBion Biotechnology Co., Ltd. (Nanjing, Jiangsu, China). All mice shared the same C57BL/6J genetic background and were male. The A53T mouse model was generated by inserting the human α-syn gene carrying the SNCA A53T mutation into the mouse genome to mimic the pathological features of familial early-onset Parkinson’s disease. The mice were randomly assigned and housed in high-altitude (Shigatse, 3,800 m) or low-altitude (Chongqing, 300 m) regions from 4 to 10 months of age, at which time the following behavioral tests were conducted. Body weight did not differ between groups before behavioral testing. All behavioral scoring and histological analyses were performed by experimenters blinded to group allocation.

Traction test: Mice were suspended by their forelimbs on a horizontal thin wire and scored as follows: 3 points if both hind limbs grasped the wire, 2 points if one hind limb grasped the wire, 1 point if neither hind limb grasped the wire but the mouse maintained grip without falling, and 0 points if it fell. Each mouse was tested three times with 2-minute intervals between trials, and the average score was calculated.

Climbing-pole test: Mice were placed head-up at the top of a vertical rough-surfaced wooden pole (50 cm in length, 1 cm in diameter) and allowed to descend autonomously. The mice were trained for two consecutive days, with each training session consisting of three trials. The time taken to turn around and reach the bottom of the pole was recorded.

Hanging endurance test (grid test): Mice were placed upward on a grid (5 mm aperture). After horizontal shaking, the grid was inverted. Once they flipped, the mice were hung upside down by their paws until they dropped to the ground. The latency to fall was recorded.

All experiments involving mice were approved by the Laboratory Animal Welfare and Ethics Committee of the Third Military Medical University (AMUWEC20224926) and were performed in accordance with the national and institutional Guidelines for the Care and Use of Laboratory Animals.

### Protein detection

Peripheral blood was collected from the mice into EDTA-coated anticoagulant tubes and centrifuged at 2,000 × g for 10 min to obtain the plasma. The mice were anesthetized and euthanized, and brain tissue was collected for staining, homogenate preparation, and omics analysis.

Brain tissues were mechanically homogenized in RIPA buffer supplemented with a protease and phosphatase inhibitor cocktail. The homogenate was ultracentrifuged to collect the supernatant as the soluble fraction. Protein concentrations were determined using the BCA protein quantification assay. Plasma and brain homogenates were analyzed by ELISA as previously described. Additionally, brain homogenates were subjected to Western blotting for target protein analysis (anti-α-syn: 1:1,000, Biolegend, 834301; anti-α-syn (phospho-S129): 1:1,000, Abcam, ab168381; anti-Claudin-5: 1:1,000, Invitrogen, 34-1600). After incubation with IRDye 800CW anti-mouse or anti‑rabbit secondary antibodies (1:5,000, LICORbio), membranes were imaged using an Odyssey system.

### Transcriptome and proteome analysis

Transcriptomics: mRNA was isolated from brain tissue samples of four mice per group and reverse-transcribed to generate double-stranded cDNA. After adapter ligation, the cDNA underwent purification, size selection, and amplification. Sequencing was performed on the DNBSEQ-T7 platform. The sequencing error rate was below 1.5%. Differential gene expression analysis was conducted with DESeq2, with screening thresholds set at |log2FC| ≥ 1 and a *p* < 0.05.

Proteomics: Four mice per group were used, and the brain tissue of each mouse was processed individually as an independent biological replicate. Proteins were extracted, reduced with tris (2‑carboxyethyl) phosphine, alkylated with iodoacetamide, diluted with triethylammonium bicarbonate, and digested with trypsin and recombinant Lys‑C in a Barocycler (Pressure BioSciences, USA). After quenching with trifluoroacetic acid, peptides were desalted on SOLAµ SPE 96‑well plates, dried, reconstituted, and quantified at 280 nm. These four biological replicates were processed in two batches, with two biological replicates per batch. In each batch, technical replicates and pooled quality control samples were included to monitor batch stability and technical reproducibility. Each biological replicate was analyzed by liquid chromatography–mass spectrometry using a Vanquish Neo UHPLC system coupled to an Orbitrap Astral mass spectrometer (Thermo Scientific, San Jose, USA). Raw DIA data were processed with DIA‑NN (v1.8.1) in match‑between‑runs mode against the UniProt reviewed mouse reference proteome (UP000000589; downloaded 19 June 2023; 17,268 proteins) supplemented with common contaminants. Search parameters included: one missed cleavage, peptide length 7–30 amino acids, precursor charge 1–4, precursor m/z 300–1800, fragment m/z 200–1800; oxidation of methionine (variable) and carbamidomethylation of cysteine (fixed). The precursor‑level false discovery rate was controlled at 1%. Proteins with a fold change > 1.5 and a *p* < 0.05 were considered statistically significant.

Pathway enrichment analysis: For transcriptomics, differentially expressed genes were subjected to Kyoto Encyclopedia of Genes and Genomes (KEGG) pathway enrichment analysis and Gene Ontology (GO) annotation analysis. For proteomics, differential proteins were analyzed by GO and WikiPathway enrichment. All enrichment analyses were performed using clusterProfiler (R version 4.0).

### Immunohistochemistry and immunofluorescence staining

Brain tissues were fixed in 4% paraformaldehyde for 24 h at 4 °C, and then dehydrated in 30% sucrose for 24 h. The tissues were sectioned into 30-µm-thick serial sections using a freezing microtome. The sections were incubated in 3% hydrogen peroxide for 10 min to quench endogenous peroxidase activity. The sections were subsequently blocked with 3% bovine serum albumin (BSA) at room temperature for 30 min and incubated overnight at 4 °C with a primary antibody (anti-α-syn (phospho-S129): 1:200, Abcam, ab184674). After the slides were rinsed three times with PBS, they were incubated with the HRP-conjugated anti-mouse IgG antibodies (Beyotime) for 1 h. The signals were developed with a DAB staining kit.

For dual immunofluorescence staining, the sections were first incubated with anti-α-syn (phospho-S129) and anti-tyrosine hydroxylase (TH) (1:200, Abcam, ab137869) primary antibodies overnight, followed by a mixture of Alexa Fluor 488-conjugated anti-mouse and Alexa Fluor 594-conjugated anti-rabbit secondary antibodies (Abcam). Nuclei were counterstained with DAPI. All stained images were captured with a Zeiss microscope. ROIs were defined based on anatomical landmarks, and the positively stained area was thresholded and expressed as a percentage of the total ROI area. Analyses were performed using Image J. All staining and detection steps were carried out according to the manufacturers’ instructions and to protocols previously established by our group [16, 17].

### Isolation and injection of erythrocyte‑derived extracellular vesicles

Total extracellular vesicles (EVs) were isolated from the plasma of PD patients using the exoEasy Maxi Kit (QIAGEN, 76064) according to the manufacturer’s instructions. To enrich erythrocyte‑derived EVs, the total EV preparation was incubated with Dynabeads (Thermo Fisher, 14311D) conjugated with an anti‑Glycophorin A antibody (Abcam, ab129024). After magnetic separation, the bound erythrocyte‑derived vesicles were eluted and labeled with the BioTracker Far Red Exosome Membrane Labeling Kit (Sigma‑Aldrich, SCT266). The labeled vesicles were resuspended in sterile PBS and injected into three-month-old male Wt C57BL/6J mice via the tail vein. Control mice received an equal volume of PBS that had been incubated with the same labeling kit under identical conditions. At 24 h post‑injection, in vivo fluorescence imaging was performed using a Newton 7.0 smart imaging system (Vilber, France) with an exposure time of 1 s. Immediately after imaging, brains were removed and processed for immunofluorescence staining and ELISA of brain homogenates as described above. In this vesicle experiment the donors were PD patients rather than high-altitude residents, the recipient wild-type mice were not exposed to altitude, and the 24-hour endpoint captured vesicle entry rather than pathology.

### Statistical analysis

The statistical analyses were performed with GraphPad Prism, version 8.0 (GraphPad Software, USA). Data were expressed as the mean ± standard deviation (SD) unless otherwise stated. A two-tailed independent t test was used for normally distributed data, whereas nonnormally distributed data were analyzed using the Mann‒Whitney U test after the Kolmogorov‒Smirnov test. Categorical variables were assessed using chi-square tests. Partial correlation analyses were performed to assess the associations between erythrocyte indices and SN hyperechogenicity while controlling for age, sex, disease duration, LEDD, and H&Y stage. For all other correlation analyses, partial correlations adjusted for age and sex were used. Two-sided *p* values less than 0.05 were considered statistically significant.

## Results

### PD prevalence in a high-altitude population and motor deficits in high-altitude A53T mice

To investigate the potential impact of high-altitude hypoxia on the progression of PD, we initially conducted an epidemiological survey among middle-aged and elderly residents (≥ 45 years) in Shigatse, Tibet (3,800 m). The flow chart outlining the process for including and excluding patients was displayed in Supplementary Fig. 1. The study subjects (*n* = 665) had comparable demographic characteristics (Supplementary Table 1).

**Fig. 1:**
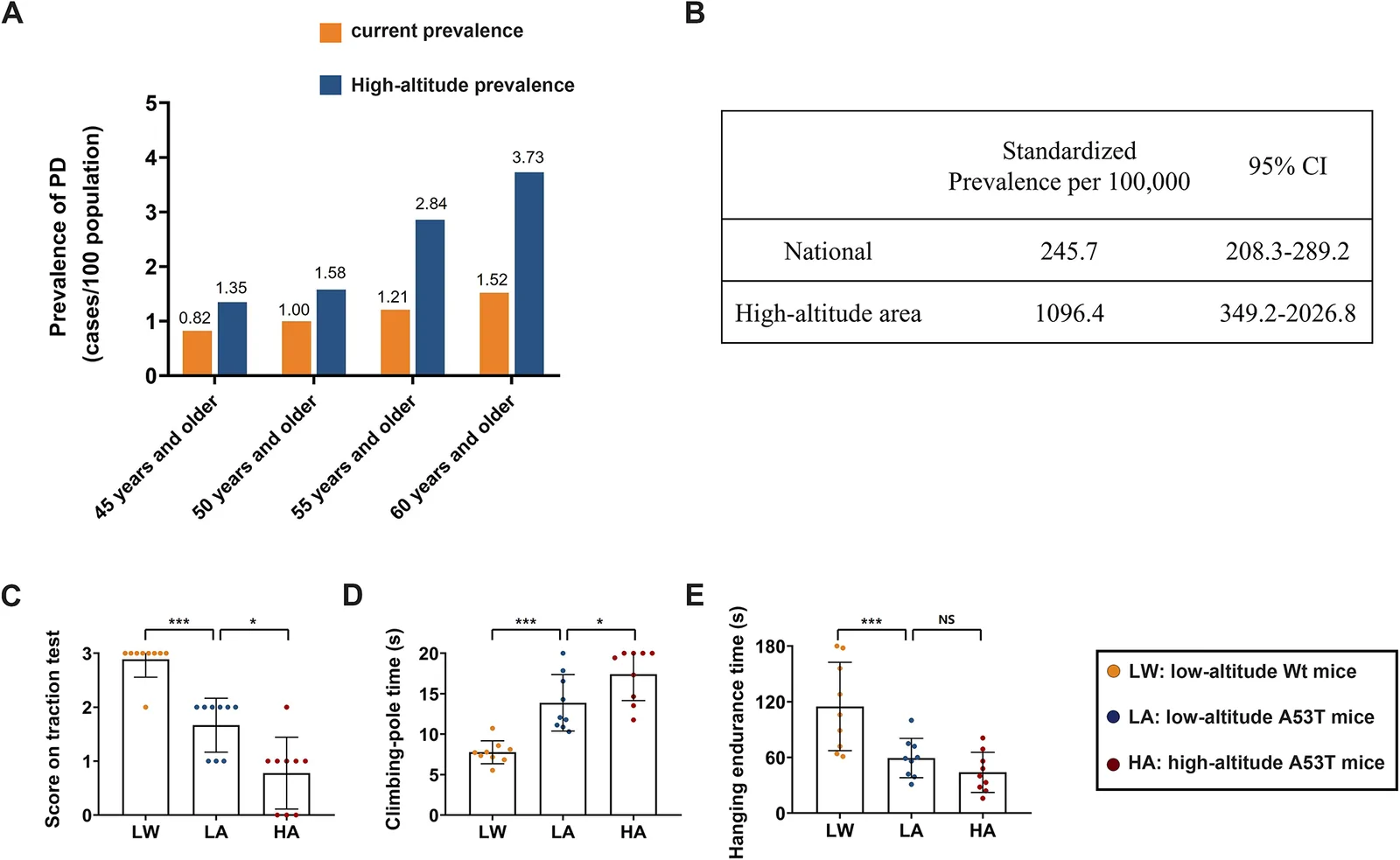
Prevalence of PD in populations and behavioral assays in mice at high altitudes. **A** Prevalence of PD in high-altitude populations compared with national averages. **B** Age-standardized prevalence of PD (≥ 40-year standard population) in the high-altitude population versus the national all-age estimate. **C‒E** Comparisons of traction (**C**), climbing-pole (**D**) and hanging endurance (**E**) tests in LW, LA and HA mice (*n* = 9 per group). LW, low-altitude Wt mice; LA, low-altitude A53T mice; HA, high-altitude A53T mice. *** denotes *p* < 0.001. * denotes *p* < 0.05. NS denotes no statistical significance. A two-tailed independent t test was used for normally distributed data, whereas nonnormally distributed data were analyzed via the Mann‒Whitney U test after the Kolmogorov‒Smirnov test was performed

Our cross‑sectional survey estimated the point prevalence of PD in a high‑altitude population. Among 665 screened participants aged ≥ 45 years, 9 were diagnosed with PD, yielding an overall prevalence of 1.35% (95% CI: 0.71%–2.55%), which tended to be higher than China’s national average of 0.82% [18]. Age‑stratified analysis revealed prevalence estimates of 1.58% (7/442; 95% CI: 0.77%–3.18%) in those aged ≥ 50 years, 2.84% (6/211; 95% CI: 1.31%–6.06%) in those aged ≥ 55 years, and 3.73% (5/134; 95% CI: 1.60%–8.44%) in those aged ≥ 60 years (Fig. 1A). After direct age standardization to the 2020 Chinese census population within the ≥ 40‑year age strata, the age‑standardized prevalence was 1096.4 per 100,000 (95% CI: 349.2–2026.8), which was numerically higher than the national all‑age estimate of 245.7 per 100,000 reported in the China Parkinson’s disease report, 2025 (Fig. 1B) [19]. However, the two estimates are not directly comparable, as the national report does not provide age‑standardized prevalence data specific to the population aged 40 years and older. Notwithstanding this limitation, these findings suggest that chronic high‑altitude residence may be associated with a higher prevalence of PD.

To investigate the effects of altitude, we maintained 4-month-old A53T transgenic mice under high-altitude and low-altitude control conditions. At 10 months, A53T mice exhibited significant motor impairments compared with wild-type controls, with high-altitude A53T mice showing exacerbated deficits. Compared with their low-altitude counterparts, high-altitude A53T mice showed more severe behavioral deficits, with significantly poorer performance in the traction test (0.78 ± 0.67 vs. 1.67 ± 0.50, *p* = 0.018) and climbing-pole test (17.41 ± 3.27 s vs. 13.87 ± 3.49 s, *p* = 0.041). Although not statistically significant, a decreasing trend in hanging endurance duration was observed in high-altitude A53T mice (43.89 ± 21.60 s vs. 59.33 ± 21.18 s, *p* = 0.145) (Fig. 1C-E). These results suggest that chronic hypoxic exposure may accelerate Parkinsonian motor dysfunction.

### Brain omics analyses suggest blood-brain barrier disruption at high altitude

**Fig. 2:**
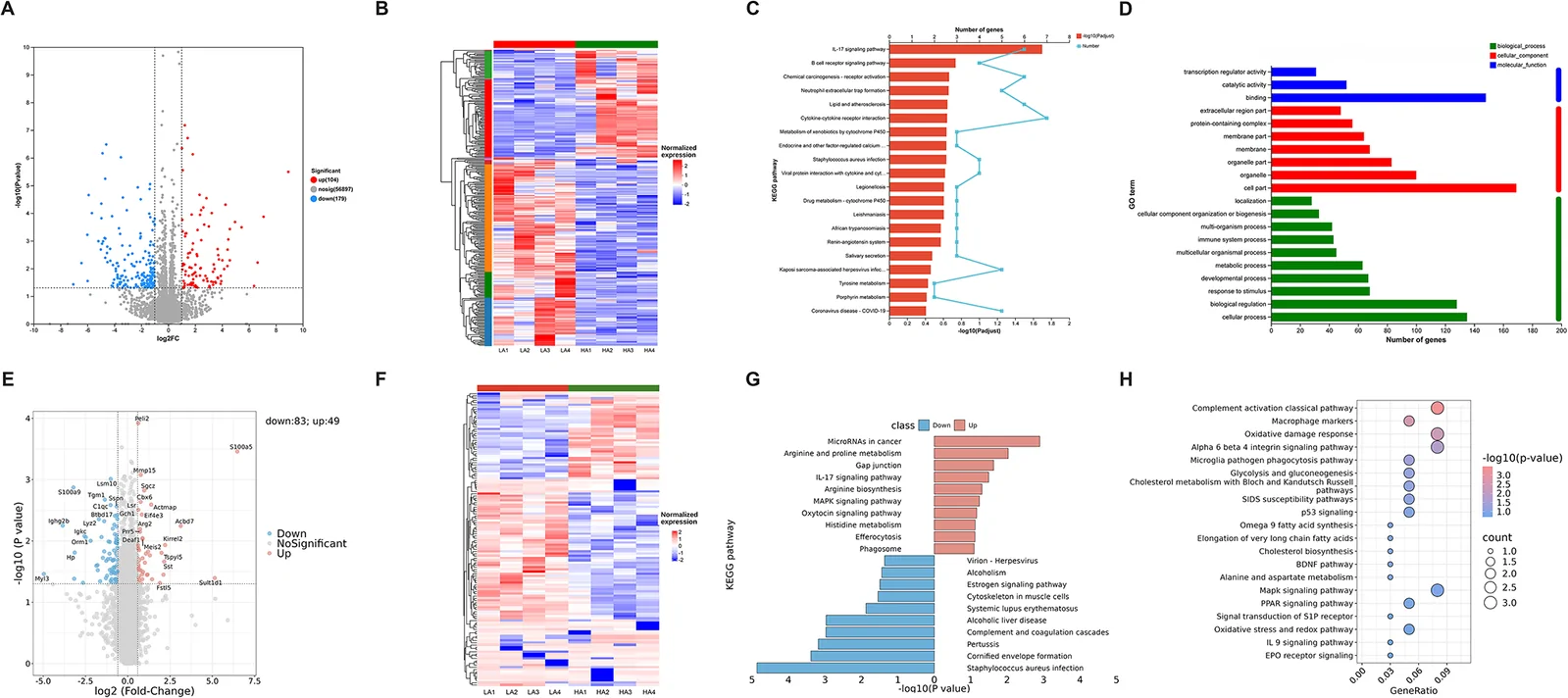
Comparisons of omics analysis in LA and HA mice.** A-D** Volcano plot (**A**), clustering heatmap (**B**), KEGG enrichment analysis (**C**) and GO annotation analysis (**D**) of differentially expressed genes between the LA and HA groups (*n* = 4). **E‒H** Volcano plot (**E**), clustering heatmap (**F**), GO (**G**) and Wikipathway (**H**) enrichment analysis of differentially expressed proteins between the LA and HA groups (*n* = 4). LA, low-altitude A53T mice; HA, high-altitude A53T mice; GO, Gene Ontology; KEGG, Kyoto Encyclopedia of Genes and Genomes

Further transcriptomic sequencing of both the high-altitude and low-altitude A53T mouse groups revealed 30,738 genes expressed in brain tissue. Compared with low‑altitude A53T mice, high‑altitude A53T mice showed 104 upregulated and 179 downregulated genes (Fig. 2A; Supplementary Table 2). The clustering heatmap of the differentially expressed genes was provided in Fig. 2B. KEGG enrichment analysis revealed significant enrichment of pathways such as IL-17 signaling and immune processes (Fig. 2C). Furthermore, GO functional annotation of differentially expressed genes highlighted alterations in enriched categories, including membrane part and cellular process (Fig. 2D). IL-17 can increase the permeability of endothelial cell membranes in the blood-brain barrier (BBB), which may disrupt barrier integrity in high-altitude mice [20].

Proteomic sequencing initially identified 11,596 peptide matches, which were filtered to 9,878 unique proteins after removing those that mapped to multiple proteins. Compared with the low-altitude A53T mice, the high-altitude A53T mice presented 49 upregulated and 83 downregulated proteins (Fig. 2E; Supplementary Table 3). A clustering heatmap of the differential proteins was shown in Fig. 2F. GO enrichment analysis revealed significant alterations in pathways related to the IL-17 signaling pathway and gap junction (Fig. 2G), highlighting the critical role of BBB disruption in high-altitude PD-related pathology. As shown by the bubble plot in Fig. 2H, Wikipathway analysis further confirmed the enrichment of pathways related to oxidative damage, inflammatory responses, and the sphingosine-1-phosphate (S1P) signaling pathway. Taken together, these findings collectively suggest that the synergistic dysregulation of immune-inflammatory responses and oxidative stress may contribute to BBB impairment in high-altitude PD pathogenesis. Emerging evidence indicates that peripheral α-syn may play a crucial role in the pathogenesis of PD, potentially serving as a seed that initiates the disease [4, 21].

### Elevated peripheral α-syn at high altitude may be associated with PD pathogenesis

**Fig. 3:**
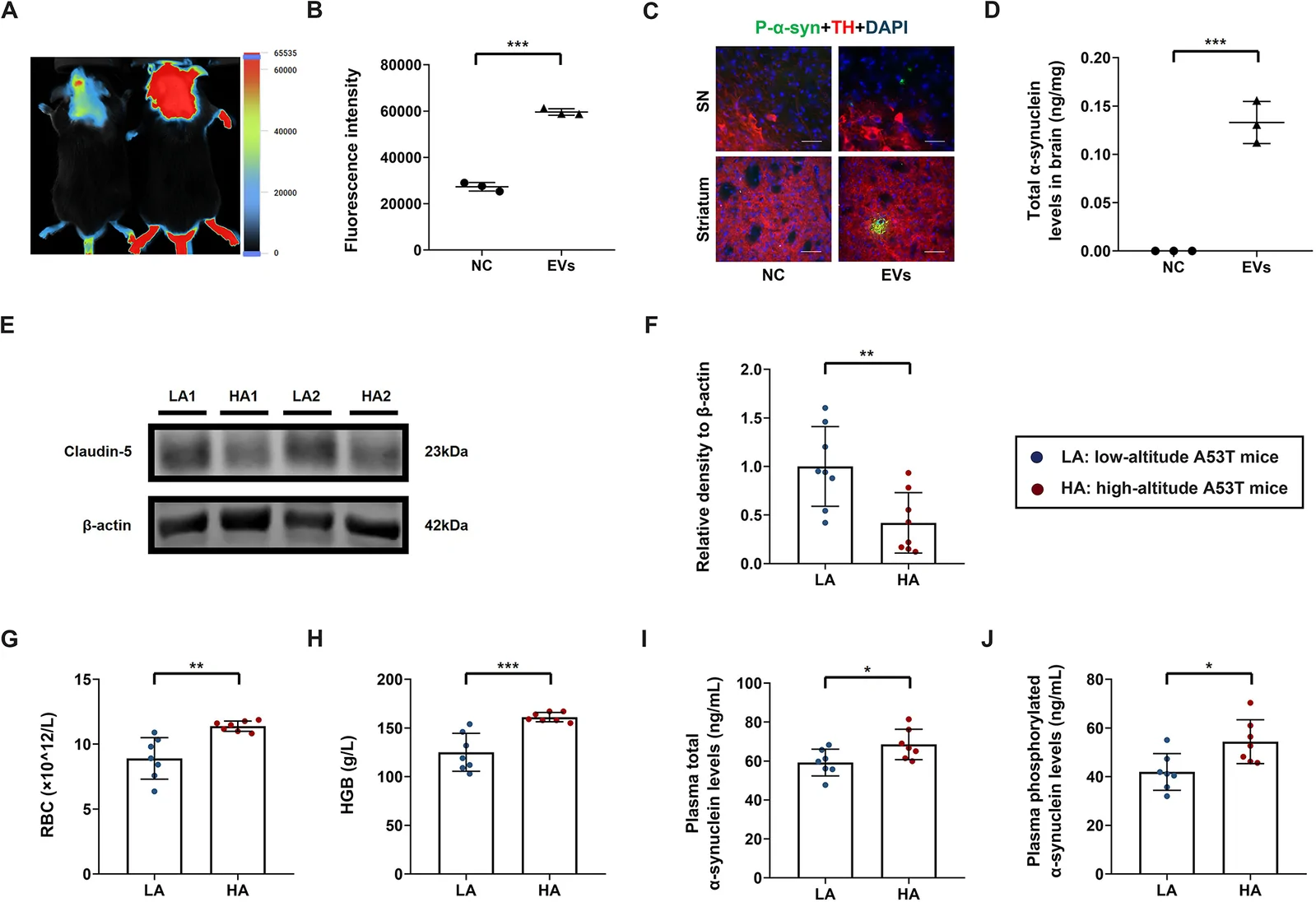
Erythrocyte-derived vesicles reaching brain tissue, blood-brain barrier integrity and peripheral blood indices in mice. **A**,** B** In vivo fluorescence imaging showing differential brain signals between the NC and EVs groups 24 h after intravenous injection (*n* = 3). **C** Comparisons of p-α-syn, TH, and DAPI staining in the SN and striatum between the NC and EVs groups. Scale bars = 200 μm. **D** Comparisons of total α-syn levels in brain homogenates between the NC and EVs groups (*n* = 3). **E**,** F** Western blot analyses of Claudin-5 in the brain homogenates of LA and HA mice (*n* = 8 per group). **G**,** H** Comparisons of RBC and HGB in LA and HA mice (*n* = 7). **I**,** J** Comparisons of plasma total α-syn and p-α-syn levels in mice in the LA and HA (*n* = 7). LA, low-altitude A53T mice; HA, high-altitude A53T mice; RBC, red blood cell count; HGB, hemoglobin; α-syn, alpha-synuclein; p-α-syn, phosphorylated alpha-synuclein; NC, normal control Wt mice; EVs, Wt mice injected with erythrocyte‑derived extracellular vesicles; TH, tyrosine hydroxylase; DAPI, 4′,6-diamidino-2-phenylindole; SN, substantia nigra. A two-tailed independent t test was used for normally distributed data, whereas nonnormally distributed data were analyzed via the Mann‒Whitney U test after the Kolmogorov‒Smirnov test was performed. *** denotes *p* < 0.001. ** denotes *p* < 0.01. * denotes *p* < 0.05

To demonstrates only that erythrocyte-derived vesicles can reach brain tissue, fluorescence‑labeled erythrocyte‑derived vesicles from PD patients were injected into wild‑type mice via the tail vein. At 24 h post‑injection, in vivo fluorescence imaging showed significantly enhanced brain fluorescence signals in the vesicle group compared with controls (Fig. 3A, B). Immunofluorescence staining of brain sections collected after transcardial perfusion detected human‑derived p‑α‑syn signals in both the periphery of SN and striatal regions (Fig. 3C). ELISA analysis of brain homogenates further detected human‑derived α‑syn in the vesicle group, whereas levels in control mice were below the assay detection limit (Fig. 3D). These findings demonstrate that peripheral erythrocyte‑derived vesicles can cross the blood‑brain barrier and enter the brain parenchyma.

Western blot analysis revealed that Claudin-5 levels were significantly decreased in HA group compared with LA group (0.41 ± 0.31 vs. 1.00 ± 0.41, *p* = 0.007) (Fig. 3E, F). Building on findings of BBB disruption in high-altitude-exposed mice, we further analyzed peripheral red cell indices and plasma α-syn levels in these mice. High-altitude mice presented significantly increased erythrocyte counts (11.39 ± 0.40 × 10^12/L vs. 8.91 ± 1.60 × 10^12/L, *p* = 0.002) and hemoglobin levels (161.1 ± 4.81 g/L vs. 125.0 ± 19.48 g/L, *p* < 0.001) (Fig. 3G, H). Additionally, the plasma levels of total α-syn (68.53 ± 7.79 ng/mL vs. 59.22 ± 6.85 ng/mL, *p* = 0.035) and p-α-syn (54.36 ± 9.01 ng/mL vs. 41.93 ± 7.56 ng/mL, *p* = 0.016) were markedly elevated in high-altitude mice compared with low-altitude controls (Fig. 3I, J). These findings suggest that peripheral α-syn dysregulation may play a role in PD pathogenesis under chronic hypoxia at high altitude.

### Elevated peripheral α-syn levels in high-altitude control individuals

**Fig. 4:**
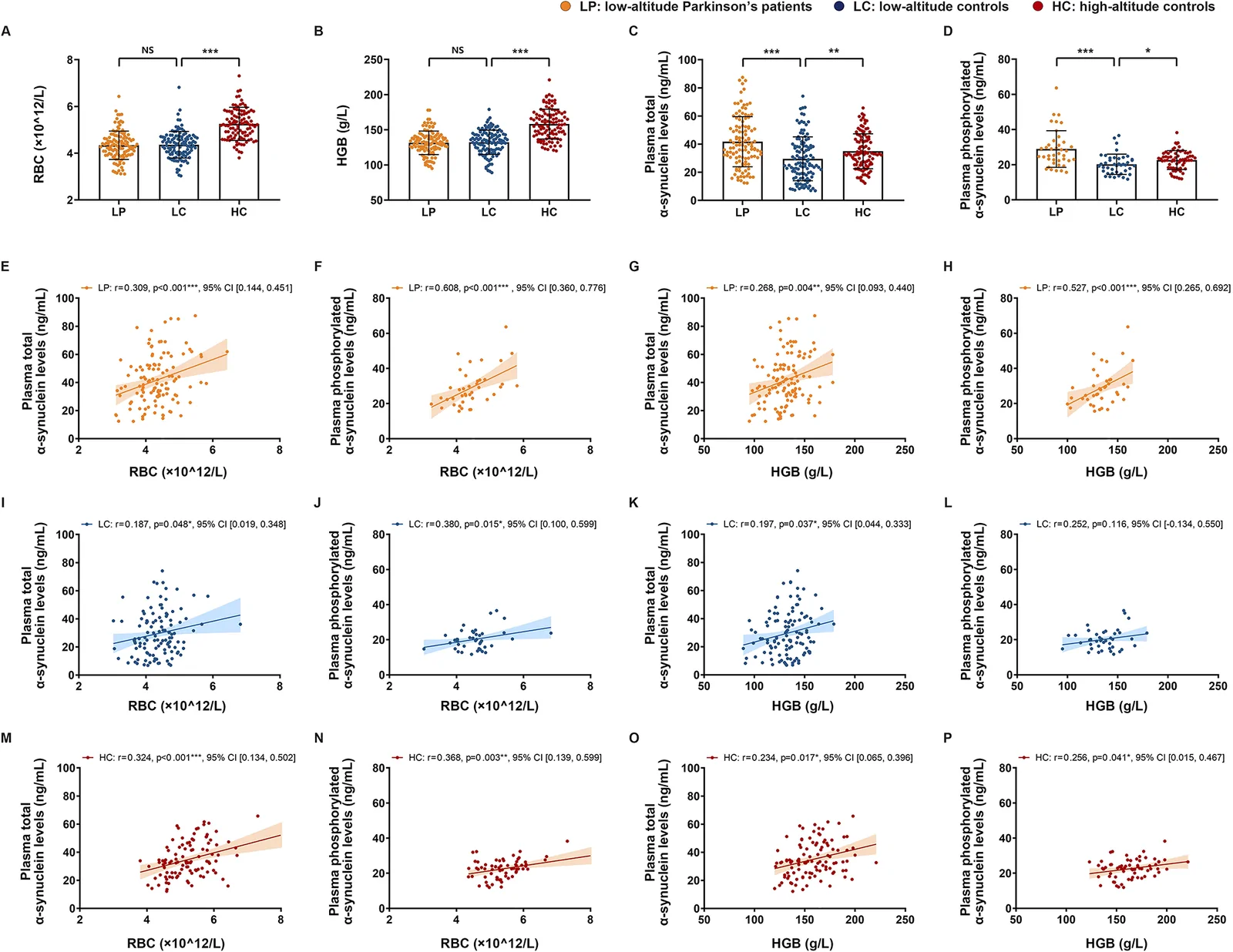
Comparisons and correlations of red cell indices with plasma α-syn levels in the LP, LC and HC populations.** A**,** B** Comparisons of RBC (A) and HGB (B) in the LP, LC and HC populations. **C**,** D** Comparisons of plasma total α-syn (**C**) and p-α-syn (**D**) levels in the LP, LC and HC populations. **E-H** Correlations of red cell indices with plasma α-syn levels in populations of LP. **I‒L** Correlations of red cell indices with plasma α-syn levels in populations of patients with LC. **M‒P** correlations of red cell indices with plasma α-syn levels in HC populations. LP, low-altitude Parkinson’s disease patients; LC, low-altitude controls; HC, high-altitude controls; RBC, red blood cell count; HGB, hemoglobin; α-syn, alpha-synuclein; p-α-syn, phosphorylated alpha-synuclein; CI, confidence interval. A two-tailed independent t test was used for normally distributed data, whereas nonnormally distributed data were analyzed via the Mann‒Whitney U test after the Kolmogorov‒Smirnov test was performed. Partial correlation analyses were adjusted for age and sex. The shaded areas represent the 95% CI. *** denotes *p* < 0.001. ** denotes *p* < 0.01. * denotes *p* < 0.05. NS denotes no statistical significance

To investigate the impacts of altitude on peripheral α-syn, we recruited low-altitude PD patients (LP), low-altitude controls (LC), and high-altitude controls (HC). The demographic parameters (sex, age, comorbidities) were not significantly different among the three groups (Supplementary Table 4). As shown in Fig. 4A, B, high-altitude individuals presented higher erythrocyte counts (5.26 ± 0.72 × 10¹²/L vs. 4.36 ± 0.57 × 10¹²/L, *p* < 0.001) and hemoglobin levels (158.2 ± 21.23 g/L vs. 132.3 ± 17.24 g/L, *p* < 0.001) than LC individuals did, while no such differences were detected between the two low-altitude groups. Moreover, significantly greater plasma levels of both total α-syn (34.93 ± 12.41 ng/mL vs. 29.53 ± 15.61 ng/mL, *p* = 0.002) and p-α-syn (22.64 ± 5.37 ng/mL vs. 20.22 ± 5.77 ng/mL, *p* = 0.029) were detected in high-altitude participants than in LC participants (Fig. 4C, D), which is consistent with the animal results.

We analyzed correlations between erythrocyte indices and α-syn levels in all groups (Fig. 4E‒P). These correlation analyses were exploratory and were not adjusted for multiple comparisons; p values are therefore descriptive. In LP patients, erythrocyte counts (α-syn: *r* = 0.309, *p* < 0.001; p-α-syn: *r* = 0.608, *p* < 0.001) and hemoglobin (α-syn: *r* = 0.268, *p* = 0.004; p-α-syn: *r* = 0.527, *p* < 0.001) were positively correlated with plasma α-syn levels. The HC group showed similar correlations between erythrocyte indices and total α-syn (RBC: *r* = 0.324, *p* < 0.001; HGB: *r* = 0.234, *p* = 0.017) as well as p-α-syn (RBC: *r* = 0.368, *p* = 0.003; HGB: *r* = 0.256, *p* = 0.041). In the LC group, weaker or non‑significant correlations were observed for RBC (α‑syn: *r* = 0.187, *p* = 0.048; p‑α‑syn: *r* = 0.380, *p* = 0.015) and HGB (α‑syn: *r* = 0.197, *p* = 0.037; p‑α‑syn: *r* = 0.252, *p* = 0.116). These exploratory findings suggest that altitude-induced polycythemia may be associated with higher α-syn levels and may contribute to PD pathogenesis.

### Erythrocyte pool expansion as the main contributor to increased peripheral α-syn burden at high altitude

**Fig. 5:**
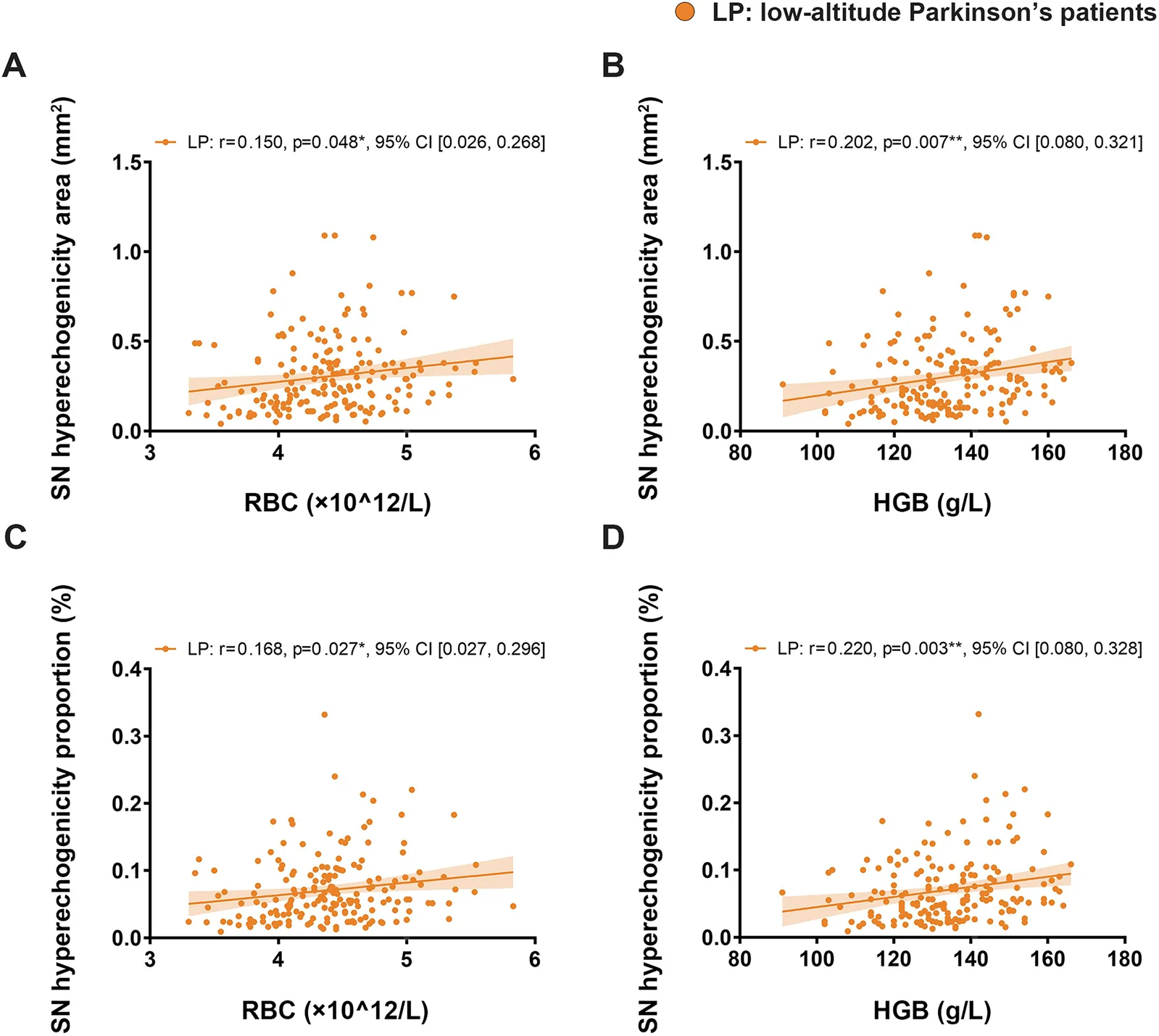
Correlations of red cell indices with SN hyperechogenicity and comparisons of erythrocytic α-syn levels. **A**,** B** Correlations of RBC (**A**) and HGB (**B**) with the SN hyperechogenic area in populations of LPs. **C**,** D** Correlations of RBC (**C**) and HGB (**D**) with the proportion of SN hyperechogenicity in populations of LP. LP, low-altitude Parkinson’s disease patients; RBC, red blood cell count; HGB, hemoglobin; SN, substantia nigra; CI, confidence interval. A two-tailed independent t test was used for normally distributed data, whereas nonnormally distributed data were analyzed via the Mann‒Whitney U test after the Kolmogorov‒Smirnov test was performed. Partial correlation analyses were adjusted for age, sex, disease duration, LEDD, and H&Y stage. The shaded areas represent the 95% CI. * denotes *p* < 0.05

We analyzed the correlations between erythrocyte indices and nigral ultrasonographic features in PD patients to further investigate erythrocyte-related effects (Supplementary Table 5). RBC showed weak positive correlations with the hyperechoic area (*r* = 0.150, *p* = 0.048) and with its proportion (*r* = 0.168, *p* = 0.027) (Fig. 5A, C). Similar associations were observed for HGB (area: *r* = 0.202, *p* = 0.007; proportion: *r* = 0.220, *p* = 0.003) (Fig. 5B, D). These findings indicate that erythrocytes may modestly influence nigral changes associated with PD progression.

**Fig. 6:**
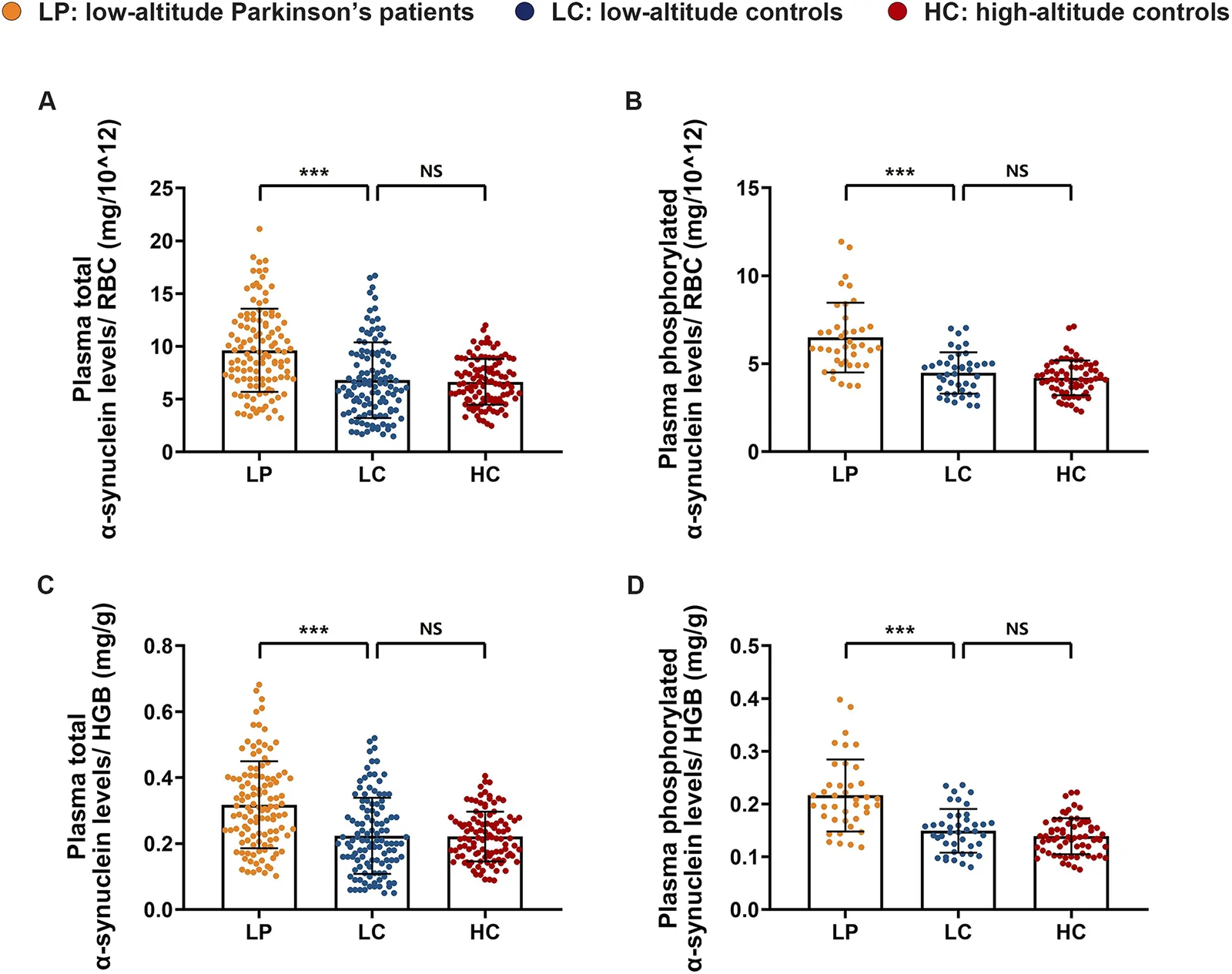
Comparisons of normalized erythrocytic α‑synuclein levels in the LP, LC and HC groups.** A**,** B** Comparisons of total α‑syn/RBC (**A**) and p‑α‑syn/RBC (**B**) in the LP, LC and HC groups. **C**,** D** Comparisons of total α‑syn/HGB (**C**) and p‑α‑syn/HGB (**D**) in the LP, LC and HC groups. LP, low-altitude Parkinson’s disease patients; LC, low-altitude controls; HC, high-altitude controls; RBC, red blood cell count; HGB, hemoglobin; α-syn, alpha-synuclein; p-α-syn, phosphorylated alpha-synuclein. A two-tailed independent t test was used for normally distributed data, whereas nonnormally distributed data were analyzed via the Mann‒Whitney U test after the Kolmogorov‒Smirnov test was performed. *** denotes *p* < 0.001. NS denotes no statistical significance

Furthermore, we isolated erythrocytes from high-altitude and low-altitude populations, with no significant differences in demographic characteristics or comorbidities between the two groups (Supplementary Table 6). Compared with low-altitude controls, high-altitude individuals presented significantly elevated erythrocyte levels of both total α-syn (25.85 ± 8.03 ng/mg vs. 18.95 ± 6.60 ng/mg, *p* = 0.036) and p-α-syn (18.16 ± 6.70 ng/mg vs. 12.12 ± 4.55 ng/mg, *p* = 0.021) in erythrocytes (Supplementary Fig. 2A, B). Given that hemoglobin dominates erythrocyte lysate protein, per‑protein values may be confounded. We therefore normalized α‑syn levels to RBC count and hemoglobin. After normalization, neither total α‑syn per 10⁶ RBC nor per gram of hemoglobin differed significantly between the LC and HC groups, although normalized α‑syn remained elevated in LP patients (Fig. 6). Consequently, the higher whole‑blood erythrocytic α‑syn burden in the high‑altitude group is attributable primarily to polycythemia, rather than to increased α‑syn content per erythrocyte. These data indicate that chronic hypoxia elevates the total peripheral α‑syn burden mainly by expanding the red blood cell pool.

### Increased brain α-syn and aggravated PD-related neuropathology in high-altitude A53T mice

**Fig. 7:**
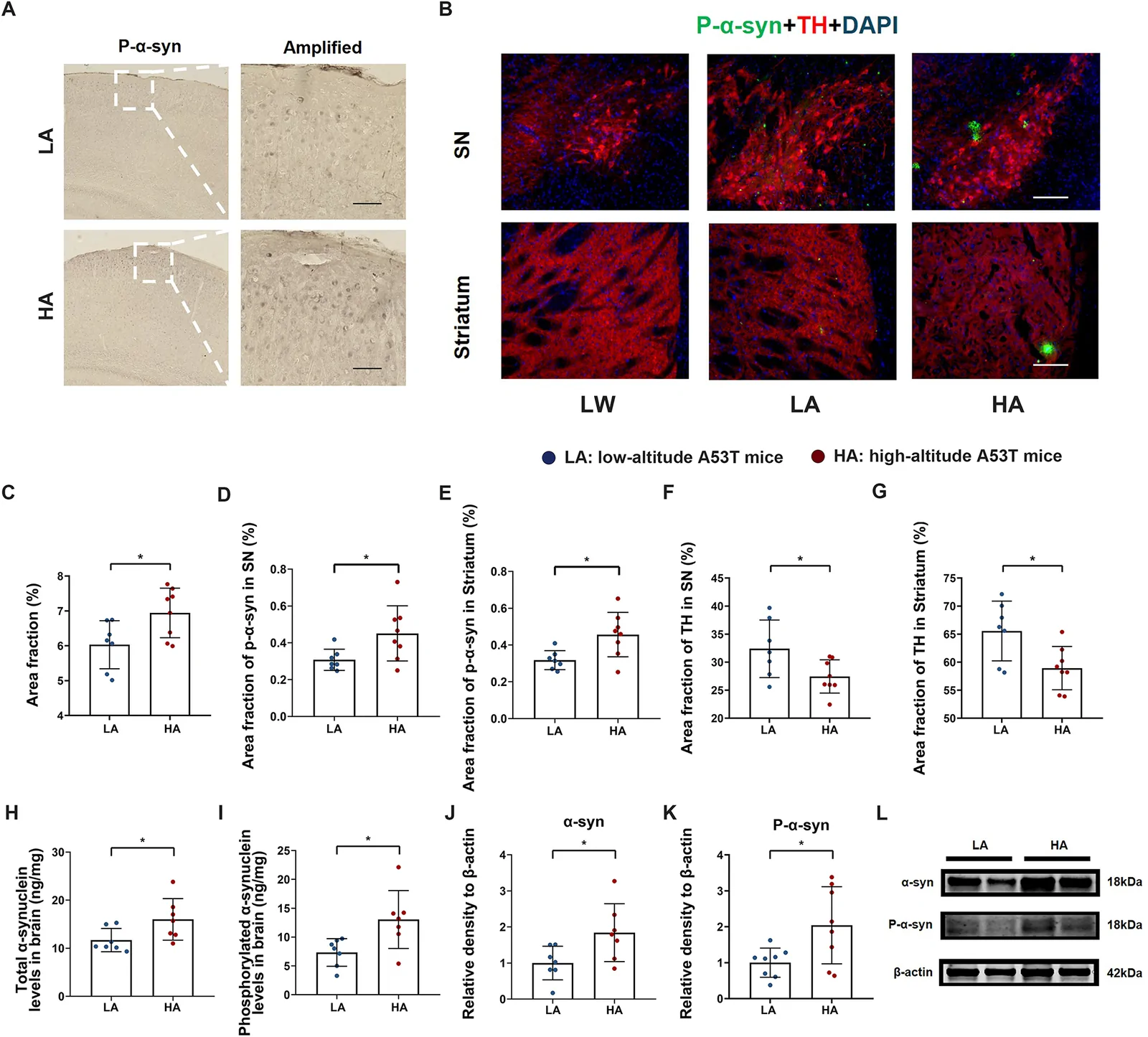
Comparisons of brain PD-related pathology in mice.** A**,** C** Comparisons of the area fraction of cells containing p-α-syn stained in the neocortex of the LA and HA groups (*n* = 7–8 per group). Scale bars = 50 μm. **B**,** D-G** Comparisons of the area fractions of p-α-syn, TH, and DAPI staining in the SN and striatum of the LA and HA groups (*n* = 7–8 per group). Scale bars = 100 μm. **H**,** I** Comparisons of total α-syn (**H**) and p-α-syn (**I**) levels in brain homogenates from mice in the LA and HA groups (*n* = 7 per group). **J‒L** Western blot analyses of total α-syn and p-α-syn in the brain homogenates of LA and HA mice (*n* = 7 per group). LW, low-altitude Wt mice; LA, low-altitude A53T mice; HA, high-altitude A53T mice; α-syn, alpha-synuclein; p-α-syn, phosphorylated alpha-synuclein; TH, tyrosine hydroxylase; DAPI, 4′,6-diamidino-2-phenylindole; SN, substantia nigra. A two-tailed independent t test was used for normally distributed data, whereas nonnormally distributed data were analyzed via the Mann‒Whitney U test after the Kolmogorov‒Smirnov test was performed. * denotes *p* < 0.05. NS denotes no statistical significance

As α-syn aggregation constitutes a core neuropathological hallmark of PD, we quantified cerebral α-syn levels in A53T transgenic mice exposed to high-altitude hypoxia. A53T mice at high altitude presented elevated total α-syn and p-α-syn levels in the brain, as shown by ELISA (α-syn: 16.01 ± 4.33 ng/mg vs. 11.69 ± 2.43 ng/mg, *p* = 0.040; p-α-syn: 13.04 ± 5.02 ng/mg vs. 7.33 ± 2.39 ng/mg, *p* = 0.019) (Fig. 7F, G). Western blot analysis supported these findings (α-syn: 1.84 ± 0.80 vs. 1.00 ± 0.47, *p* = 0.034; p-α-syn: 2.04 ± 1.07 vs. 1.00 ± 0.41, *p* = 0.022) (Fig. 7J-L). Immunohistochemical analysis revealed increased p-α-syn deposition (6.94 ± 0.71% vs. 6.03 ± 0.69%, *p* = 0.026) (Fig. 7A, C), whereas immunofluorescence revealed expanded p-α-syn-positive areas in the SN (0.45 ± 0.15% vs. 0.31 ± 0.058%, *p* = 0.034) and striatal regions (0.46 ± 0.12% vs. 0.32 ± 0.052%, *p* = 0.015) (Fig. 7B, D, E). Notably, no p-α-syn staining was observed in LW mice, confirming the human specificity of the antibody under our experimental conditions. Tyrosine hydroxylase immunostaining revealed aggravated dopaminergic neuron loss in high-altitude mice within the SN (27.45 ± 2.96% vs. 32.39 ± 5.13%, *p* = 0.037) and striatum (58.94 ± 3.86% vs. 65.57 ± 5.33%, *p* = 0.019), consistent with exacerbated neurodegeneration under high-altitude chronic hypoxia (Fig. 7H, I). These findings further demonstrate that increasing peripheral erythrocytic α-syn at high altitudes may contribute to PD-related neuropathology in the brain.

## Discussion

This study provides evidence that high-altitude environments may be associated with a higher prevalence of PD. According to classical theory, this association may stem from neuronal intolerance to chronic hypoxic conditions. Dopaminergic neurons in the substantia nigra extend long-range, highly branched axonal projections to the striatum, resulting in substantial energy requirements and a high demand for oxygen and ATP. This anatomical and metabolic vulnerability renders them particularly susceptible to oxidative stress and hypoxic damage [22]. Notably, our findings show that hypoxic conditions are associated with higher levels of brain and peripheral α-syn, accompanied by increased brain α-syn deposition and dopaminergic neuron loss. These results suggest a new mechanistic connection between chronic hypoxia and PD pathogenesis.

As a key pathological driver of PD, α-syn aberrantly accumulates within neurons [3]. Pathologically phosphorylated α-syn contributes significantly to neurodegeneration by inducing mitochondrial dysfunction and synaptic deficits, ultimately leading to dopaminergic cell loss [23]. Previous studies have suggested that hypoxia promotes the expression and phosphorylation of α-syn, potentially accelerating PD pathogenesis [24]. Our descriptive proteomic profiling of A53T mouse brain indicated enrichment of S1P signaling. S1P has been shown to inhibit PP2A activity, thereby impairing p‑α‑syn clearance [25, 26]. We therefore speculate that chronic high‑altitude exposure could compromise α‑syn degradation via an S1P‑PP2A‑mediated mechanism. This impaired clearance of PD-related pathological proteins would in turn be expected to aggravate central degeneration, which is consistent with our animal experiments.

In addition to its direct effects on the central nervous system, high-altitude hypoxia can also raise the total peripheral α-syn burden. This occurs primarily through a compensatory increase in erythrocytes, thereby expanding the primary cellular source of α-syn [27]. Our data support this mechanism in both the animal experiments and the human cohort, and provide no evidence that high altitude increases α-syn content per erythrocyte. Although the relative contributions of peripheral versus central α-syn to high-altitude PD remain incompletely understood, increasing evidence indicates that peripherally derived α-syn can enter the brain and may contribute to disease progression [28].

Our study further suggests that high-altitude environments can compromise BBB integrity, potentially facilitating the entry of erythrocyte-derived α-syn into the central nervous system and contributing to neurodegeneration [21]. In this process, IL-17, which is upregulated under plateau conditions, may play a role [29, 30]. These peripheral erythrocyte-derived α-syn molecules can translocate into the brain and contribute to central neurodegeneration under conditions of compromised BBB integrity, potentially establishing a vicious cycle that may accelerate the pathogenesis of PD [21, 31]. Consequently, the combined effects of hypoxia and compensatory erythrocytosis may facilitate the accumulation of peripherally derived α-syn in the central nervous system, advancing PD progression. While a recent study suggested that several weeks of hypoxia may be protective in a PFF‑based PD model [32], our study differs in several key aspects. We employ hypobaric hypoxia in a real high‑altitude environment, used A53T transgenic mice, and conducted prolonged observation, together with a preliminary epidemiological survey. These differences may explain the divergent outcomes.

One of the primary limitations of this study is our inability to precisely quantify the contribution of peripheral α-syn to PD pathogenesis under high-altitude conditions, underscoring the need for further mechanistic investigation. The epidemiological component employed a cross‑sectional design with a relatively small cohort from extreme plateaus. We were unable to adjust for potential confounders such as ethnicity, rurality, occupation, or smoking, which limits causal inference and may compromise generalizability. Larger longitudinal studies across varying altitudes are warranted. Owing to practical challenges in high-altitude settings, we were unable to perform CSF α-syn detection or DAT-PET imaging, or direct assessment of blood-brain barrier integrity in this cohort, which limited further insight into PD-related central neurodegeneration under these conditions. We also acknowledge that behavioral tests may be influenced by hypoxia and fatigue inherent to the high‑altitude environment. The inclusion of a high‑altitude Wt group in future studies would be valuable to distinguish from environment‑specific effects. It should be noted that α-syn measured in A53T mouse plasma and brain with human-specific kits is transgene-derived, and its regulation need not mirror that of endogenous human erythroid α-syn. Additionally, hemolysis was assessed only by visual inspection, and no quantitative free hemoglobin or absorbance index was available. Its impact on plasma α‑syn measurements remains a concern. Future studies should incorporate quantitative hemolysis indices to account for this. The correlation analyses were exploratory and unadjusted for multiple comparisons; these findings should therefore be regarded as hypothesis-generating rather than confirmatory. Finally, while our findings suggest a role for erythrocyte-derived α-syn in PD progression, direct interventional evidence is still lacking, representing an important avenue for future investigation.

## Conclusions

Our research suggests that high-altitude environment may increase both central and peripheral α-syn levels, an effect likely mediated by chronic hypoxia-induced erythropoiesis, ultimately contributing to the pathogenesis of PD. These findings underscore the association between prolonged high-altitude exposure and the risk of neurodegenerative disorders.

## Supplementary Information

Below is the link to the electronic supplementary material.

Supplementary Material 1

Supplementary Material 2

Supplementary Material 3

## Data Availability

The data of this study contained in the manuscript being submitted have not been previously published or submitted elsewhere. The data are available from the corresponding author upon reasonable request.

## Abbreviation

α-syn: Alpha-synuclein
BBB: Blood-brain barrier
BCA: Bicinchoninic acid
CI: Confidence interval
COPD: Chronic obstructive pulmonary disease
DAB: Diaminobenzidine
DAPI: 4′,6-diamidino-2-phenylindole
EDTA: Ethylenediaminetetraacetic acid
ELISA: Enzyme-linked immunosorbent assay
EVs: Extracellular vesicles
GO: Gene Ontology
HA: High-altitude A53T mice
HC: High-altitude controls
HGB: Hemoglobin
H&Y: Hoehn & Yahr
KEGG: Kyoto Encyclopedia of Genes and Genomes
LA: Low-altitude A53T mice
LC: Low-altitude controls
LEDD: Levodopa-equivalent daily dose
LP: Low-altitude Parkinson’s disease patients
LW: Low-altitude wild-type mice
OSAS: Obstructive sleep apnea syndrome
PBS: Phosphate-buffered saline
PD: Parkinson’s disease
PP2A: Protein phosphatase 2A
P-α-syn: Phosphorylated α-syn
RBC: Red blood cell count
RIPA: Radio Immunoprecipitation Assay
S1P: Sphingosine-1-phosphate
SD: Standard deviation
SN: Substantia nigra
SpO₂: Peripheral oxygen saturation
TCS: Transcranial sonography
TH: Tyrosine hydroxylase
Wt: Wild type
