# Supplementary material for: High-altitude environments elevate central and peripheral α-synuclein levels and accelerate PD-related pathology
Source: J Transl Med. 2026 Oct 21;24:1376. doi: 10.1186/s12967-026-09103-z (PMC13723142; doi:10.1186/s12967-026-09103-z)
Supplement: Supplementary file 1 [file 12967_2026_9103_MOESM1_ESM.docx]

**High-altitude environments elevate central and peripheral α-synuclein levels accelerate PD-related pathology**

Hao-Lun Sun ^1, 2, 3 #^, Dong-Yu Fan ^1, 2, 3 #^, Jun-Hao Xie ^2, 3, 4 #^, Qing Du ^4^, Rui-Jie Zeng ^1^, Jian-Ni Hu ^5^, Jun Liang ^5^, Li Zhu ^5^, Xu Wang ^5^, Hao-Xiang Wang ^6^, Qing Zhao ^1, 7^, Rong Zhang ^8^, Yan-Jiang Wang ^1, 2, 3^, Hong-Da Zhao ^5 *^, Wang-Sheng Jin ^1, 2, 3 *^

^1^ Department of Neurology and Centre for Clinical Neuroscience, Daping Hospital, Third Military Medical University, Chongqing, China.

^2^ Chongqing Key Laboratory of Ageing and Brain Diseases, Chongqing, China.

^3^ Institute of Brain and Intelligence, Third Military Medical University, Chongqing, China.

^4^ Department of Special Medicine, Daping Hospital, Third Military Medical University, Chongqing, China.

^5^ Shigatse Branch, Xinqiao Hospital, Third Military Medical University, Shigatse, China.

^6^ Department of Neurology, Xinqiao Hospital & The Second Affiliated Hospital, Third Military Medical University, Chongqing, China.

^7^ Department of Neurology, The 910th Hospital of the Joint Logistics Support Force of the Chinese PLA, Quanzhou, Fujian Province, China.

^8^ Department of Neurology, Army 73rd group military hospital of the Chinese PLA, Xiamen, Fujian Province, China.

^#^ These authors contributed equally to this work.

^*^ Correspondence to Dr. Wang-Sheng Jin (jinwangsheng@tmmu.edu.cn). Address: No. 10 Changjiang Branch Road, Yuzhong District, Chongqing, China. Telephone and fax: +86 23 68729550.

Or correspondence to Mr. Hong-Da Zhao (497631818@qq.com). Address: No. 5 Zhufeng West Road, Shigatse, Tibet Autonomous Region, China. Telephone and fax: +86 0892 68819608.

**Supplementary Table legends**

**Supplementary Table 1** **Characteristics of the study subjects with epidemiological investigation in high altitude.**

| **Characteristics** | **n=665** |
| --- | --- |
| Age, mean (SD), y | 53.67 (7.37) |
| Female, n (%) | 222 (33.38) |
| Hypertension, (%) | 65 (9.77) |
| Diabetes, (%) | 23 (3.46) |
| Dyslipidemia, (%) | 66 (9.92) |

SD, standard deviation.

**Supplementary Table 2 Differentially expressed genes of high-altitude and low-altitude A53T mice.** See accompanying Excel file.

**Supplementary Table 3** **Differentially expressed proteins of high-altitude and low-altitude A53T mice.** See accompanying Excel file.

**Supplementary Table 4** **Characteristics of the study subjects with plasma between LP, LC and HC.**

| **Characteristics** | **LP (n=117)** | **LC (n=115)** | **HC (n=106)** | ***P1* value (LP vs. LC)** | ***P2* value (LC vs. HC)** |
| --- | --- | --- | --- | --- | --- |
| Age, mean (SD), y | 63.97 (8.09) | 62.49 (10.35) | 60.74 (8.58) | 0.137 | 0.225 |
| Female, n (%) | 46 (39.32) | 51 (44.35) | 48 (45.71) | 0.437 | 0.839 |
| Duration high-altitude time, mean (SD), y | - | - | 47.52 (21.90) | - | - |
| Actual altitude, mean (SD), y | - | - | 3938 (341.5) | - | - |
| SpO₂, mean (SD), % | - | - | 87.34 (4.08) | - | - |
| Hypertension, mean (SD), % | 40 (34.19) | 28 (24.35) | 27 (25.47) | 0.100 | 0.847 |
| Diabetes, mean (SD), % | 17 (14.53) | 16 (13.91) | 10 (9.43) | 0.893 | 0.302 |
| Dyslipidemia, mean (SD), % | 25 (21.37) | 33 (28.70) | 22 (20.75) | 0.198 | 0.173 |

LP, low-altitude Parkinson's patients; LC, low-altitude controls; HC, high-altitude controls; SD, standard deviation; SpO₂, peripheral oxygen saturation. A two-tailed independent t-test was utilized to compare data with a normal distribution, whereas the Mann–Whitney U test was employed for non-normal data. Percentage data used the chi-square test.

**Supplementary Table 5** **Characteristics of the study subjects with substantia nigra ultrasound in LP.**

| **Characteristics** | **n=181** |
| --- | --- |
| Age, mean (SD), y | 66.89 (8.49) |
| Female, n (%) | 51 (38.06) |
| Hypertension, (%) | 33 (24.63) |
| Diabetes, (%) | 14 (10.45) |
| Dyslipidemia, (%) | 31 (23.13) |
| Disease duration, mean (SD), y |  |
| LEDD, mean (SD), mg |  |
| H&Y stage, mean (SD) |  |
| RBC, mean (SD), ×10^12/L | 4.39 (0.48) |
| HGB, mean (SD), g/L | 133.6 (15.24) |
| SN hyperechogenicity area, mean (SD), mm^2^ | 0.29 (0.19) |
| SN hyperechogenicity proportion, mean (SD), % | 0.066 (0.043) |

LP, low-altitude Parkinson's patients; SD, standard deviation; RBC, red blood cell counts; HGB, hemoglobin; SN, substantia nigra; LEDD, Levodopa-equivalent daily dose; H&Y, Hoehn & Yahr.

**Supplementary Table 6** **Characteristics of the study subjects with erythrocytes between LP and LC.**

| **Characteristics** | **LC (n=11)** | **HC (n=12)** | ***p* value** |
| --- | --- | --- | --- |
| Age, mean (SD), y | 65.18 (9.23) | 62.67 (7.75) | 0.661 |
| Female, n (%) | 4 (40.00) | 3 (25.00) | 0.452 |
| Duration high-altitude time, mean (SD), y | - | 62.67 (7.75) | - |
| Actual altitude, mean (SD), y | - | 3875 (160.3) | - |
| SpO₂, mean (SD), % | - | 88 (1.71) | - |
| Hypertension, (%) | 0 (0) | 1 (8.33) | 0.350 |
| Diabetes, (%) | 0 (0) | 0 (0) | >0.999 |
| Dyslipidemia, (%) | 2 (20.00) | 4 (33.33) | 0.484 |
| RBC, mean (SD), ×10^12/L | 4.32 (0.26) | 5.21 (0.42) | <0.001 |
| HGB, mean (SD), g/L | 133.5 (7.54) | 160.8 (17.38) | <0.001 |

LC, low-altitude controls; HC, high-altitude controls; SD, standard deviation; RBC, red blood cell counts; HGB, hemoglobin. Two-tailed independent t-tests, Mann-Whitney U test or chi-squared test was used as appropriate.

**Supplementary Figure legends**

**Supplementary Figure 1 Recruitment flowcharts. A** Flowchart of the epidemiological investigation group in Shigatse. **B** Flowchart of the group with plasma. **C** Flowchart of the group with Transcranial Sonography. **D** Flowchart of the group with erythrocyte collection.

**Supplementary Figure 2 Comparisons of erythrocytic α-syn levels. A, B** Comparisons of erythrocytic total α-syn (A) and p-α-syn (B) levels in the LC and HC populations. LC, low-altitude controls; HC, high-altitude controls; α-syn, alpha-synuclein; p-α-syn, phosphorylated alpha-synuclein. A two-tailed independent t test was used for normally distributed data, whereas nonnormally distributed data were analyzed via the Mann‒Whitney U test after the Kolmogorov‒Smirnov test was performed. * denotes p < 0.05.
